# Keys to an Early Diagnosis of Miller Fisher Syndrome: A Case of Miller Fisher Syndrome Masquerading as Myasthenia Gravis

**DOI:** 10.7759/cureus.84834

**Published:** 2025-05-26

**Authors:** Chamine Robince, Kinnari Modi, Peter Park, Tara Norris

**Affiliations:** 1 Internal Medicine, Methodist Health System, Dallas, USA; 2 Internal Medicine, Burnett School of Medicine, Texas Christian University, Dallas, USA

**Keywords:** areflexia, emg, gbs, mfs, myasthenia gravis, ophthalmoplegia, sensory changes

## Abstract

Miller Fisher syndrome (MFS) and myasthenia gravis can present with similar symptoms. However, some key differences can help differentiate between these two disorders. Early identification is extremely important for MFS so treatment can be initiated in a timely manner. Here, we present the case of a 33-year-old female who was initially diagnosed with myasthenia gravis and was later found to have MFS. The goal of this case report is to provide information to aid in the early diagnosis and identification of MFS.

## Introduction

Miller Fisher syndrome (MFS) is a rare variant of Guillain-Barré Syndrome (GBS) that affects 1 to 2 people per 1,000,000 people worldwide [1]. MFS is observed in about 1% to 5% of GBS cases in Western countries [2]. MFS patients typically present with a triad of symptoms: ophthalmoplegia, ataxia, and areflexia. However, some patients do not demonstrate the full triad of symptoms at initial presentation. Similar to GBS, a preceding illness is identified in most MFS cases. The pathogenesis of MFS is putatively due to molecular mimicry triggered by the preceding illness. Although MFS can be self-limited, some patients have symptoms lasting six months or longer. In these cases, treatment with intravenous immunoglobulin (IVIG) is required [1]. Having a high index of clinical suspicion is required to diagnose MFS early. We present the case of a 33-year-old female who was diagnosed with MFS.

## Case presentation

A 33-year-old female with no significant past medical history presented to the emergency department with complaints of inability to move her eyes, drooping of her eyelids, and right-sided extremity weakness and tingling for five days. Further history revealed that the patient had an illness three weeks prior with sore throat, cough, and congestion that had since resolved. The patient reported that her symptoms started in the right eye and eventually progressed to the left eye. Associated symptoms included numbness of the medial three fingers of her right hand. The patient did not endorse recent fever, chills, myalgias, diplopia, eye discharge, floaters, flashes, vision loss, hearing impairment, seizures, prior head trauma, chest pain, or hemoptysis. She also denied any recent travel, gastrointestinal illness, and family history of neurological disease.

Upon evaluation, the patient’s vital signs were stable, and she was alert, conversant, and easily able to follow commands. Neurological examination revealed anisocoria, with a mydriatic right pupil (5 mm) and a normal left pupil. Absent pupillary light response was noted in the right eye; however, the left eye was minimally reactive. Examination was pertinent for near complete ophthalmoplegia of the right eye in all directions, and marked ophthalmoplegia of the left eye with abduction and downward gaze. Visual field testing was limited by severe ophthalmoplegia. Moderate partial ptosis was present bilaterally, once again with the right eye worse than the left. Sensation to light touch, temperature, and vibration were intact throughout. The patient had normal tone and 5/5 strength in all extremities, intact reflexes throughout, and normal gait. Finger-to-nose and heel-to-shin testing were normal, and she had no pronator drift. The Babinski reflex, Romberg’s and Lhermitte’s signs, and ice pack test were all negative. An ocular examination with Wood’s lamp was unrevealing, and a tonometry exam was unremarkable.

**Figure 1:**
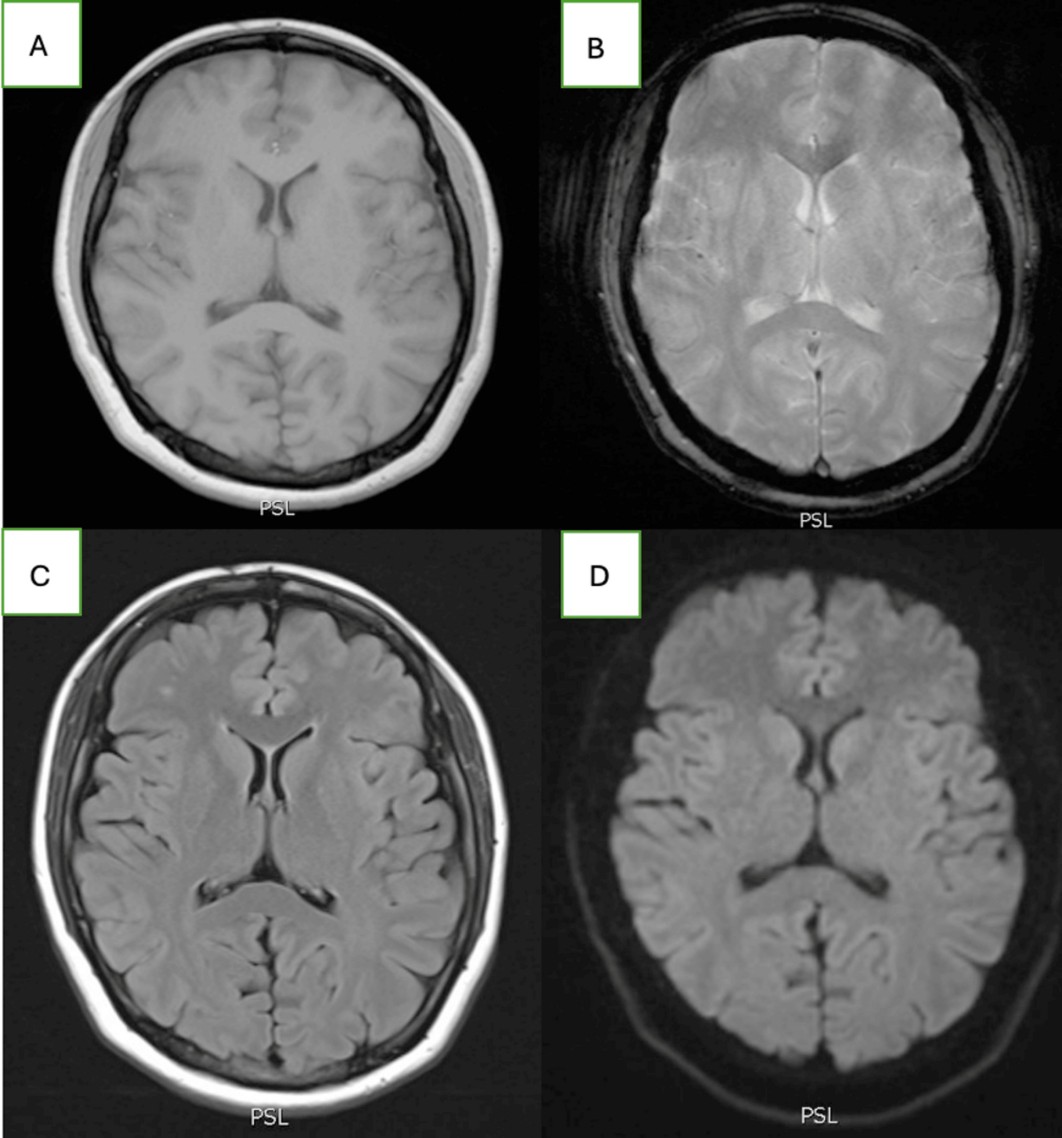
Normal MRI of the brain (A) Normal T1-weighted sequence, (B) Normal T2-weighted sequence, (C) Normal FLAIR sequence, (D) Normal diffusion-weighted imaging FLAIR: fluid-attenuated inversion recovery

Laboratory testing revealed a normal complete blood count, comprehensive metabolic panel, thyroid markers, respiratory pathogen panel, and procalcitonin level. A urine drug screen and blood ethanol levels were negative. Additional labs, such as human immunodeficiency virus (HIV), rapid plasma regain, vitamin B12, folate, and thiamine, were also negative (Table 1). Her erythrocyte sedimentation rate was mildly elevated at 46 mm/hr, and her C-reactive protein was within normal limits. Computed tomography (CT) of the head and a CT angiogram of the head and neck were negative for acute pathology or aneurysms. A CT venogram was negative for cerebral venous sinus thrombosis. Magnetic resonance imaging of the brain with contrast was unremarkable; it was without white matter lesions or periventricular lesions to suggest multiple sclerosis (Figure 1). The patient was evaluated by tele-neurology, and an initial diagnosis of ocular myasthenia gravis (OMG) was made. The patient was started on oral pyridostigmine (60 mg TID) and intravenous solumedrol (1 g daily). MFS was considered early on in the differential, and a GQ1b ganglioside antibody panel was ordered on admission.

**Table 1:** Laboratory results of the patient ALT: alanine aminotransferase; AST: aspartate aminotransferase; HIV: human immunodeficiency virus; RPR: rapid plasma reagin; ESR: erythrocyte sedimentation rate; CRP: C-reactive protein

Test	Reference Range	Results
White Blood Cells	3-10 x 10^3 ^/µL	3.4
Hemoglobin	12-16 g/dL	12.2
Hematocrit	36-46%	37.8
Platelets	130-400 x 10^3^/µL	187
Sodium	135-148 mmol/L	135
Potassium	3.4-5.1 mmol/L	4.2
Chloride	95-106 mmol/L	101
Blood Urea Nitrogen	10-25 mg/dL	14
Creatinine	0.7-1.4 mg/dL	0.7
Total Bilirubin	0.0-1.4 mg/dL	0.4
ALT	<35 U/L	23
AST	8-42 U/L	25
Alkaline Phosphatase	38-126 U/L	72
Thyroid Stimulating Hormone	0.47-4.68 uIU/mL	0.49
Procalcitonin	<0.25 ng/mL	<0.05
HIV 1&2 Antigen and Antibody	Negative	Negative
RPR	Non-Reactive	Non-Reactive
Vitamin B12	211-911 pg/mL	730
Folate	3-20 ng/mL	5.6
Vitamin B1	70-180 nmol/L	160
ESR	0-20 mm/HR	46
CRP	<10 mg/L	8

On the second hospital day, her symptoms continued to progress with worsening ptosis bilaterally, left eye mydriasis (5 mm), and complete ophthalmoplegia of the left eye. Negative inspiratory force and forced vital capacity were measured every eight hours to monitor for respiratory decline. Myasthenia gravis panel ordered on admission resulted negative (Table 2). Cerebrospinal fluid studies from a lumbar puncture performed on day three of hospitalization were non-contributory (Table 3). Electromyography (EMG) testing showed absent F wave responses in both the right upper and lower limbs. Since pupillary involvement is atypical for MG, MFS was still on the differential, and reflexes were monitored daily. On day five of hospitalization, the patient exhibited new absence of her bilateral patellar, Achilles, and triceps deep tendon reflexes. With the new areflexia on exam, progressive ophthalmoplegia, and lack of improvement with pyridostigmine and solumedrol, our patient was diagnosed with MFS. On day five of the hospitalization, she was started on five days of IVIG and had a favorable response. The ganglioside antibody panel returned strongly positive for ganglioside (GQ1b) IgG antibodies at 262 IV (normal range: 0-50 IV), supporting the diagnosis of MFS (Table 4).

**Table 2:** Myasthenia gravis (MG) panel MG laboratory tests resulted negative for the patient. MuSK: muscle-specific kinase; IgG: immunoglobulin G

Test	Reference Range	Results	Interpretation
Acetylcholine Binding Antibody	0.0-0.4 nmol/L	0.0	Negative
Acetylcholine Blocking Antibody	0-26%	26	Negative
Titin Antibody	0.00-0.45 IV	0.10	Negative
MuSK Autoantibody, Serum	0.00-0.02 nmol/L	0.00	Negative
Striated Muscle IgG Screen	<1:40	<1:40	Negative

**Table 3:** Cerebrospinal fluid analysis CSF studies were non-contributory with no evidence of CNS infection. CSF: cerebrospinal fluid; CUMM: cells per cubic millimeter; VDRL: venereal disease research laboratory; HSV: herpes simplex virus; PCR: polymerase chain reaction; RNA: ribonucleic acid

CSF Studies	Reference Ranges	Results
Appearance	Clear	Clear
Color	Colorless	Colorless
White blood cells, CSF	0-5 CUMM	1
Red blood cells, CSF	0-10 CUMM	1
Glucose, CSF	40-70 mg/dL	53
Protein, CSF	12-60 mg/dL	51
VDRL, CSF	Non-Reactive	Non-Reactive
HSV 1&2 PCR, CSR	Not Detected	Not Detected
Enterovirus RNA, Qualitative Real-Time PCR	Not Detected	Not Detected

**Table 4:** Ganglioside antibody panel results Negative: ≤29 IV; Equivocal: 30-50 IV; Positive: 51-100 IV; and Strong positive: ≥101 IV GM1: ganglioside GMI antibody; GD1B: ganglioside GD1B antibody; GQ1B: ganglioside GQ1B antibody; IgM: immunoglobulin M; IgG: immunoglobulin G

Test	Reference range	Results	Interpretation
GD1B IgG	0-50 IV	5	Negative
GM1 IgG	0-50 IV	53	Positive
GM1 IgM	0-50 IV	11	Negative
GQ1B IgG	0-50 IV	262	Strong Positive
GQ1B IgM	0-50 IV	33	Equivocal

## Discussion

Diagnosis of MFS traditionally requires two out of the three symptoms of the triad (i.e., ophthalmoplegia, ataxia, and areflexia) to be present. Multiple diagnostic tests, including lumbar puncture, can help to narrow down the differential diagnosis of MFS. Increased total protein concentration with normal total nucleated cell count (i.e., albuminocytologic dissociation) can be a hallmark feature in patients with GBS. However, only half the patients will have elevated cerebrospinal fluid (CSF) protein levels during the initial phase of illness, and about 88% of the patients can have this finding at three weeks [1]. Hegen et al. found that CSF total protein and serum quotient of albumin have low diagnostic sensitivities for GBS, particularly in the first week after disease onset [3]. Therefore, a normal protein level in CSF, especially during the early stages of the disease (week one), does not rule out MFS/GBS.

Nerve conduction studies can also aid in the diagnosis of MFS. While there is a great deal of literature examining the utility of EMG for diagnosing GBS, there is a relative paucity that is specific to MFS. A retrospective study looked at nerve conduction studies in 31 patients diagnosed with GBS. This study found that 97% of GBS patients have an absent H reflex, 84% of patients have an abnormal F wave, and 61% have a low-amplitude or absent sensory nerve action potential (SNAP) in the upper extremities [4]. EMG was used to effectively diagnose only 55% of patients. Therefore, EMG can be used to support a diagnosis of MFS, but it is not a reliable diagnostic test in and of itself. A normal EMG also cannot be used to rule out GBS or MFS.

Gangliosides are structures found on the surface of peripheral nerve cells that play crucial roles in myelination, signal transduction, and cell-to-cell communication [5]. Antiganglioside antibodies have been primarily associated with Campylobacter jejuni, which mimics gangliosides such as GM1, GD1a, and GT1 on the surface of neural tissue [6]. Studies suggest that molecular mimicry between bacteria and neural tissue results in cross-reactivity of ganglioside antibodies, such as anti-GQ1b, resulting in demyelination and axonal damage [7]. A case series in Japan found that the anti-GQ1b antibody test has a >95% sensitivity and >90% specificity for MFS [8]. Once again, a negative test does not rule out MFS. However, considering the high sensitivity and specificity, anti-GQ1b serology can be a key diagnostic marker of MFS in the appropriate clinical setting. The biggest limitation to this test is that it is typically a send-out lab that can take days to result. Therefore, a high index of suspicion is required as treatment should not be delayed while awaiting serology results.

The presence or absence of pupillary light response can aid in distinguishing between MFS and OMG. OMG can mimic MFS with partial or complete ophthalmoplegia involving the oculomotor nerve (CN III), trochlear nerve (CN IV), and the abducens nerve (CN VI). However, pupillary examination and reflexes are usually normal in OMG [9]. Although our patient’s ophthalmoplegia would fit the diagnosis of OMG, her unequal pupillary dilation and lack of pupillary light response made the initial diagnosis of OMG questionable, and ultimately supported the diagnosis of MFS. The ice pack test can also be a helpful tool in differentiating between OMG and MFS. Resolution of ptosis has been reported in >90% of OMG patients after the ice pack test [9]. According to one study, the sensitivity and specificity of this test were 76.9% and 98.3%, respectively [10]. During admission, we placed an ice pack over the patient’s closed right eyelid for about two minutes to evaluate for improvement. After the two minutes, there was no improvement or resolution of the right ptosis. An additional distinguishing factor is that the weakness and ophthalmoplegia seen with OMG may improve and change over the course of days. The symptoms in MFS will likely be persistent and continue to progress during the initial course of the illness, as seen with our patient.

The presence of right-hand numbness was another important finding that aided our clinical diagnosis. It was previously thought that MFS does not cause motor weakness or sensory deficits of the limbs [1]. However, based on the literature review, it is becoming increasingly evident that altered sensory changes and weakness may be present with MFS. There are multiple case reports documenting the involvement of cranial nerves in MFS other than the typically involved CN III, IV, and VI. It is important to recognize that atypical neurologic symptoms such as limb dysesthesia; face, bulbar, or pupillary palsies; mild motor weakness; and micturition disturbances can be seen with MFS [2].

Our patient initially presented with ophthalmoplegia along with right upper extremity paresthesias. Areflexia did not present until five days into her hospitalization, which suggests that unexplained neurological symptoms along with one of the three classic triad should raise the consideration of MFS as a differential. MFS should be higher on the differential, especially if the presenting symptom is ophthalmoplegia, as very few disorders can mimic this.

## Conclusions

It is important to recognize that the classic triad of MFS may not always be present on initial presentation. Although tests such as EMG, anti-GQ1b serology, and CSF studies can aid in the diagnosis, MFS is primarily a clinical diagnosis. Careful physical examination is critical to identify new or worsening findings, such as areflexia, ophthalmoplegia, abnormal pupillary light response, and paresthesias, which can aid in the early diagnosis of MFS.
